# Clinical characteristics and prognostic value of the *KRAS G12C* mutation in Chinese non-small cell lung cancer patients

**DOI:** 10.1186/s40364-020-00199-z

**Published:** 2020-06-25

**Authors:** Si-Yang Liu, Hao Sun, Jia-Ying Zhou, Guang-Ling Jie, Zhi Xie, Yang Shao, Xian Zhang, Jun-Yi Ye, Chun-Xiang Chen, Xu-Chao Zhang, Qing Zhou, Jin-Ji Yang, Yi-Long Wu

**Affiliations:** 1 Guangdong Lung Cancer Institute, Guangdong Provincial People’s Hospital, Guangdong Academy of Medical Sciences, School of Medicine, South China University of Technology, 106 Zhongshan Er Road, Guangzhou, 510080 China; 2 Nanjing Geneseeq Technology Inc., Nanjing, 210032 China; 3 Burning Rock Biotech, Guangzhou, 510000 China

**Keywords:** *KRAS* mutation, *KRAS G12C* mutation, Prognosis, Non-small cell lung cancer, Chinese patients

## Abstract

**Background:**

The *KRAS* mutation is the second most common genetic variant in Chinese non-small cell lung cancer (NSCLC) patients. At the 2019th World Conference of Lung Cancer, the *KRAS G12C*-specific inhibitor AMG510 showed promising results in the phase I clinical trial. However, the frequency, clinical characteristics, and prognostic significance of the *KRAS G12C* mutation in Chinese NSCLC patients are rarely reported.

**Methods:**

Next-generation sequencing was used to confirm the *KRAS* mutation status in 40,804 NSCLC patients from multiple centers (mCohort). Survival data were collected retrospectively from 1456 patients at one of the centers, the Guangdong Lung Cancer Institute (iCohort).

**Results:**

In the mCohort, 3998 patients (9.8%) were confirmed to harbor a *KRAS* mutation, of whom 1179 (29.5%) had the *G12C* subtype. In the iCohort, 130 NSCLC patients (8.9%) had a *KRAS* mutation and 42 (32.3%) had the *G12C* subtype. The *G12C* subgroup included more male patients (85.2% vs 67.4%, *P* < 0.0001) and more smokers (76.2% vs 53.4%, *P* = 0.02) than did the non-*G12C* subgroup. Both the *KRAS* mutation group and *KRAS G12C* mutation subgroup were associated with a shorter median overall survival (OS) than wildtype tumors (15.1 vs 26.7 months, hazard ratio [HR]_*KRAS*_ = 1.50, *P* = 0.002; 18.3 vs 26.7 months, HR_*G12C*_ = 1.66, *P* = 0.007). In Cox regression analysis, smoking (HR = 1.39, *P* = 0.05) and stage IV disease (HR = 2.72, *P* < 0.001) remained as independent predictors of shorter OS. Both the *KRAS* mutation (HR = 1.30, *P* = 0.07) and *KRAS G12C* mutation (HR = 1.47, *P* = 0.07) reached borderline significance.

**Conclusions:**

In the largest sample used thus for, our study found that approximately 10% of Chinese NSCLC patients had *KRAS* mutations. Of these, nearly 30% harbored the *KRAS G12C* mutation subtype, which was most common in male smokers. The *KRAS G12C* mutation is a biomarker of poor prognosis in Chinese NSCLC patients, which could potentially be improved by *G12C*-specific inhibitors in the future.

(296 words)

## Background

Rapid developments have been achieved in the area of epidermal growth factor-receptor tyrosine kinase inhibitors (EGFR-TKIs) and immunotherapy with checkpoint inhibitors for lung cancer patients [1–5]. Although treatment resistance is inevitable, advances of third-generation EGFR-TKIs prolong the survival of patients with *EGFR* mutations. The *KRAS* mutation is the second most common genetic variant in Chinese non-small cell lung cancer (NSCLC) patients [6]. Many retrospective and prospective studies have attempted to treat *KRAS* mutation patients with EGFR-TKIs [7] and MAP-ERK kinase (*MEK*) inhibitors [8, 9], but none were successful. No targeted therapy is available for patients with *KRAS* mutations and chemotherapy remains the standard. Patients with *KRAS* mutations seemed to respond to checkpoint blockade therapy in several recently published studies [10–12]. In addition to immunotherapy with checkpoint inhibitors, *KRAS G12C*-specific inhibitors show promising preclinical and clinical results [13]. The World Conference of Lung Cancer in 2019 presented promising and up-to-date clinical data on the drug AMG510, which was given to 13 patients with non-small cell lung cancer (NSCLC) at a dose of 960 mg once per day. Seven patients achieved partial response and six achieved stable disease. The objective response rate was 54% and the disease control rate was 100%. Additionally, a series of clinical trials targeting *KRAS G12C* mutations with *G12C*-specific inhibitors, including RMC-4630 and MRTX849 are ongoing [14, 15].

It was reported that 30% of Caucasian NSCLC patients harbored *KRAS* mutations, of which 35 ~ 45% were of the *G12C* subtype [16, 17]. Therefore, the incidence of *KRAS G12C* mutations in NSCLC in those Caucasian patients was nearly 12%. In Asians, however, the frequency of *KRAS G12C* mutations has rarely been studied and the prognosis of those carrying the *G12C* mutation is still unclear. Here, we examined the incidence of this mutation subtype, and its clinical characteristics, in Chinese NSCLC patients drawn from two cohorts and explored the prognostic role of the *KRAS G12*C mutation.

## Methods

### Patients

From January 2016 to September 2019, the NGS results from 40,804 NSCLC patients from multiple centers (mCohort) were analyzed; of these patients, 3998 had *KRAS* mutations. In total, 1776 patients with *KRAS* mutations had NGS results analyzed from tumor tissue, 1646 from tumor tissue and liquid biopsy, and 576 from liquid biopsy alone (e.g., peripheral blood, pleural effusion and cerebrospinal fluid). Clinical data of these patients were pooled retrospectively and the factors included in the analysis were age, sex, pathology, and clinical stage at the time of diagnosis. Smoking history and survival data of 1456 NSCLC patients from one of the centers, the Guangdong Lung Cancer Institute (iCohort), were collected retrospectively from the electronic medical records.

### Analysis

The chi-square test was used to compare categorical data. Overall survival (OS) was measured from the date of pathological diagnosis of lung cancer to the date of death or last follow-up, with a cut-off date of September 2019. Kaplan-Meier survival curves were generated to estimate OS in different genomic groups. The univariate and multivariate Cox proportional hazards model was used to evaluate the prognostic value of *KRAS* and *KRAS G12C* mutations on OS. Statistical significance was defined as a *p*-value less than 0.05.

## Results

### Frequency of KRAS G12C mutations in Chinese NSCLC patients

**Fig. 1:**
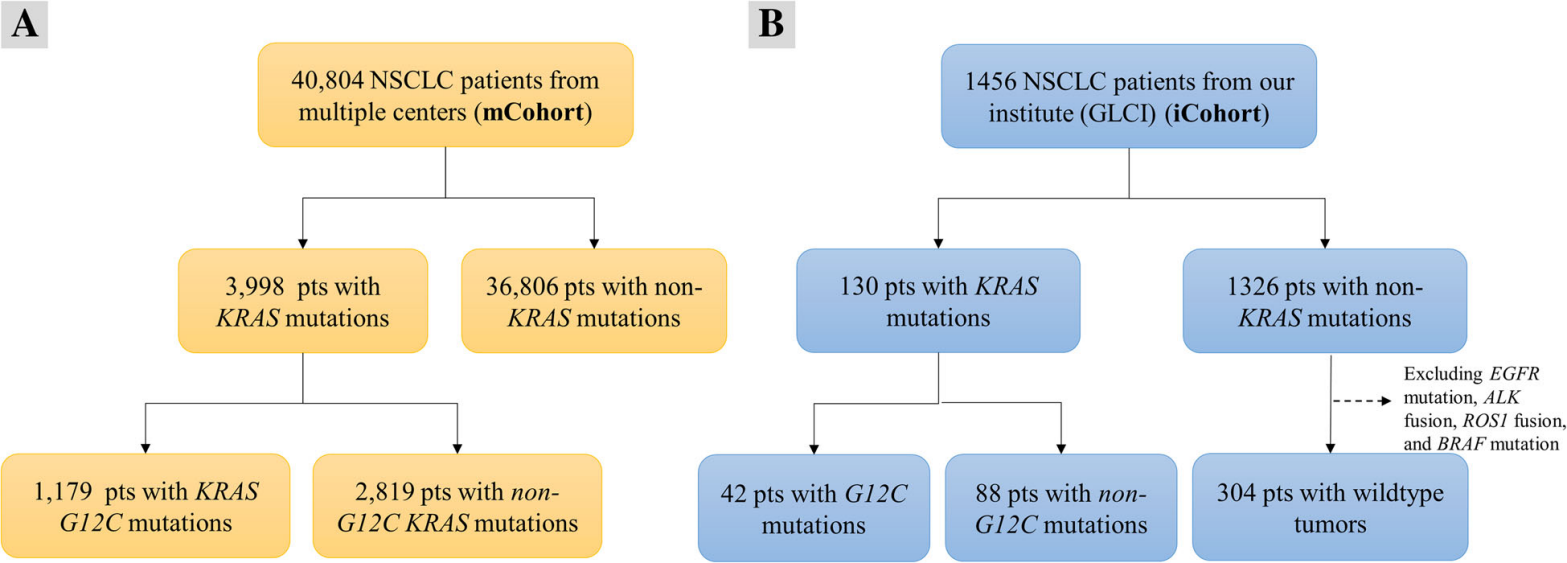
Flow charts of NSCLC patient enrollment in the study. Patients included from multiple centers cohort (mCohort) **a**. Patients included and excluded from Guangdong Lung Cancer Institute cohort (iCohort) **b**. pts.: patients

**Fig. 2:**
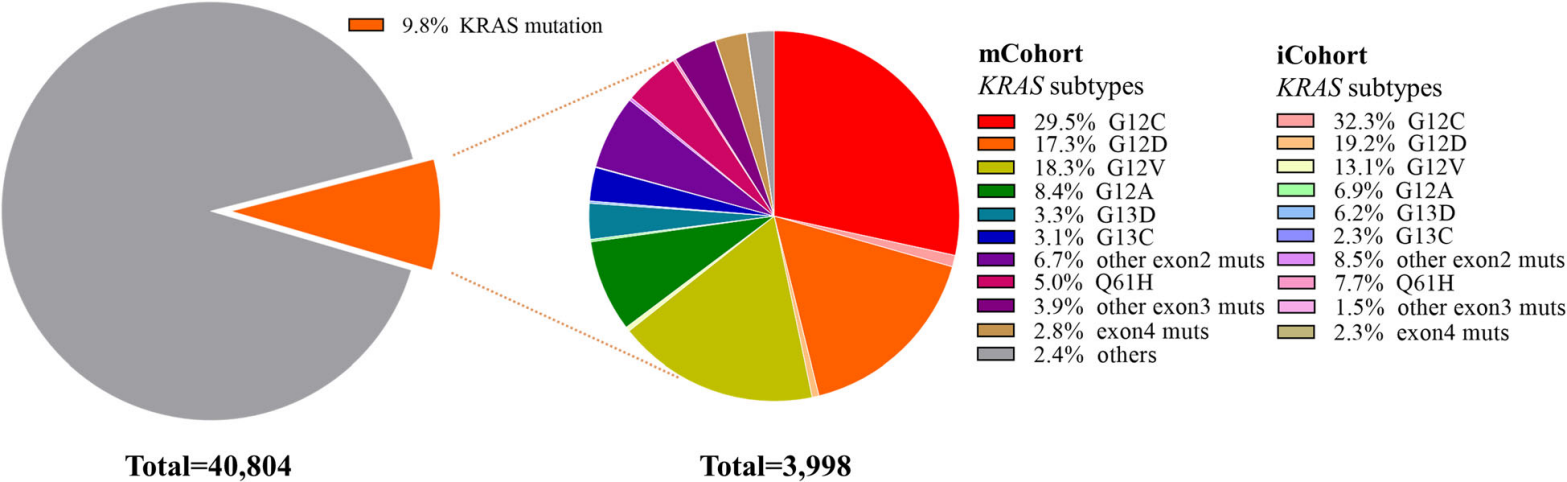
Pie charts of NSCLC patients with *KRAS* mutations. Pie charts showing the proportions of *KRAS* mutation and wildtype tumors in the mCohort (left), and the proportions of different *KRAS* mutation subtypes in the mCohort (right) which included patients from the iCohort

In the mCohort, 3998 NSCLC patients had *KRAS* mutations; 25 patients had two *KRAS* mutation subtypes, and the frequency of *KRAS* mutations was 9.8%. Of the patients with *KRAS* mutations, 1179 (29.5%) were confirmed to harbor *G12C* mutations (Fig. 1a). The proportions of the other three major *KRAS* codon 12 subtypes were as follows: *G12V*, 18.3% (*N* = 731); *G12D*, 17.3% (*N* = 693); and *G12A*, 8.4% (*N* = 334) (Fig. 2).

In the iCohort, 130 of 1456 NSCLC patients (8.9%) were confirmed to have *KRAS* mutations, of whom 42 (32.3%) harbored *G12C* mutations; there were 304 wildtype patients (excluding *EGFR* mutation, *ALK* fusion, *ROS1* fusion and *BRAF* mutation) (Fig. 1b). The distribution of *KRAS* mutation subtypes was comparable to that in the mCohort. For the other three major codon 12 subtypes, the proportions were as follows: *G12D*, 19.2% (*N* = 25); *G12V,* 13.1% (*N* = 17); and *G12A*, 6.9% (*N* = 9) (Fig. 2).

### Clinical characteristics of patients with the KRAS G12C mutation

The clinical and pathological characteristics of NSCLC patients with *KRAS G12C* and non-*G12C KRAS* mutations were compared in the mCohort (Table 1). The mean ages of the *G12C* and non-*G12C* subtype patients were 63 and 62 years old, respectively (*P* = 0.02). The proportion of male patients was higher in the *G12C* subgroup than that in the non-*G12C* subgroup (85.2% vs 67.4%, *P* < 0.0001). Most of the *G12C* and non-*G12C* subtype patients were diagnosed with adenocarcinoma (rates were more than 90% in both groups). Nearly 40% of the patients were diagnosed with stage IV disease in the *G12C* and non-*G12C* subgroups. In the iCohort, the clinical and pathological characteristics of the patients with *G12C* and non-*G12C* mutations were comparable to those in the mCohort. Of note, in the iCohort, 76.2% of the patients in the *G12C* subgroup were former or current smokers, compared with 53.4% of those in the non-*G12C* subgroup (*P* = 0.02).

**Table 1:** Clinical and pathological characteristics of *KRAS G12C* and non-*G12C* mutations from the mCohort

	*KRAS G12C* (*N* = 1179)	Non-*G12C* (*N* = 2819)	*P*-value
Age, mean (range)	63 (31–91)	62 (14–90)	0.02
Sex, *n* (%)			
Male	1005 (85.2%)	1899 (67.4%)	***
Female	162 (13.7%)	875 (31.0%)	
NA	12 (1.0%)	45 (1.6%)	
Pathology, *n* (%)			
Adenocarcinoma	1107 (93.9%)	2584 (91.7%)	#
Squamous carcinoma	23 (2.0%)	154 (5.5%)	
Adeno-squamous carcinoma	9 (0.8%)	18 (0.6%)	
LCLC	3 (0.3%)	13 (0.5%)	
others	37 (3.1%)	50 (1.8%)	
Stage, *n* (%)			
I	37 (3.1%)	85 (3.0%)	0.24
II	38 (3.2%)	86 (3.1%)	
III	125 (10.6%)	235 (8.3%)	
IV	447 (37.9%)	1082 (38.4%)	
NA	532 (45.1%)	1331 (47.2%)	

*#* could not be computed, *mCohort* lung cancer patients from multiple centers, *iCohort* lung cancer patients from Guangdong Lung Cancer Institute, *NA* not available, *LCLC* large-cell lung cancer

The clinical characteristics of *KRAS* mutation and wildtype tumors were also compared in the iCohort. In the *KRAS* mutation and wildtype subgroups, 79.2 and 71.7% of the patients were male (*P* = 0.10), respectively, with median ages of 63 and 61 years (*P* = 0.01); 60.8 and 53.3% were former or current smokers (*P* = 0.15), respectively. Most patients in both the *KRAS* mutation and wildtype subgroups had adenocarcinoma and stage IV disease.

### Prognostic value of the KRAS G12C mutation

**Fig. 3:**
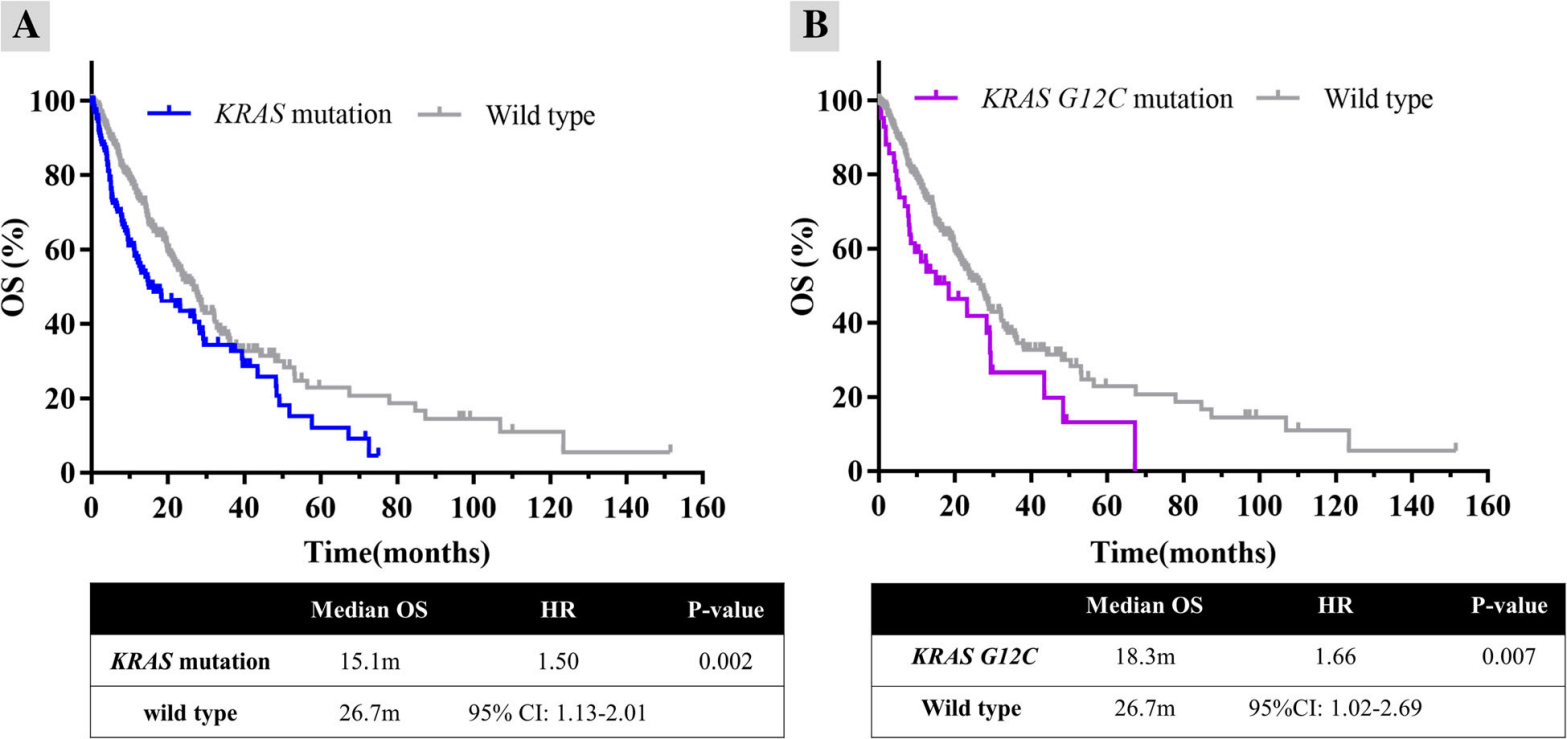
Survival analysis of NSCLC patients with *KRAS* and *KRAS G12C* mutations. Overall survival (OS) analysis of *KRAS* mutation and wildtype tumors **a**. OS analysis of *KRAS G12C* mutation and wildtype tumors **b**. m: months; wt: wild type; HR: hazard ratio; CI: confidence interval

Survival data were collected retrospectively for 130 *KRAS* mutation and 304 wildtype patients from the iCohort. In the Kaplan-Meier analysis, regardless of the *KRAS* mutation group or the *KRAS G12C* mutation subgroup, both were associated with a shorter median OS compared with wildtype tumors (15.1 vs 26.7 months, Hazard Ratio [HR]_*KRAS*_ = 1.50, *P* = 0.002; 18.3 vs 26.7 months, HR_*G12C*_ = 1.66, *P* = 0.007) (Fig. 3a, b).

To identify the prognostic values of the *KRAS* and *KRAS G12C* mutations on OS, clinical and molecular variables were included in Cox regression analysis. In the univariate analysis, age, male, smoker, stage IV disease, *KRAS* mutation, and *KRAS G12C* and non-*G12C* mutations were identified as independent factors for shorter OS (Table 2). In the multivariate Cox model, smoker (HR = 1.39, *P* = 0.05) and stage IV disease (HR = 2.72, *P* < 0.0001) remained as independent factors for poor prognosis. Both the *KRAS* mutation (HR = 1.47, *P* = 0.07) and the *KRAS G12C* mutation (HR = 1.23, *P* = 0.07) were borderline statistically significant.

**Table 2:** Univariate and multivariate analysis of overall survival based on clinical and molecular variables

Variable	Univariate analysis			Multivariate analysis		
	Crude HR	95%CI	*P*	Adjusted HR	95%CI	*P*
Age	1.02	1.00–1.03	0.02	1.01	1.00–1.03	0.07
Sex						
Female	1			1		
Male	1.41	1.05–1.90	0.02	1.08	0.74–1.58	0.71
Ever Smoking						
Non-smoker	1			1		
Smoker	1.46	1.13–1.90	0.004	1.39	1.00–1.94	0.05
Pathology						
Adenocarcinoma	1					
Squamous carcinoma	1.16	0.71–1.88	0.55			
others	1.14	0.64–2.06	0.65			
Stage						
I-II	1			1		
III	1.55	0.88–2.74	0.13	1.58	0.89–2.81	0.12
IV	2.68	1.60–4.47	***	2.72	1.62–4.56	***
*KRAS* mutation						
Wildtype	1			1		
Mutation	1.51	1.16–1.98	0.003	1.30	0.98–1.72	0.07
*KRAS* mutation subtype						
Wild-type	1			1		
*G12C* mutation	1.65	1.10–2.47	0.02	1.47	0.97–2.23	0.07
Non-*G12C* mutation	1.45	1.06–1.98	0.02	1.23	0.90–1.69	0.20

*NSCLC* non-small cell lung cancer, *HR* hazard ratio

## Discussion

The frequency of *KRAS* mutations is much higher in Caucasian NSCLC patients, at around 30%, than in Asian patients [18]. In our study, 9.8% of the patients in the mCohort harbored *KRAS* mutations, similar to the rates reported by Zhou’s group [19]. *EGFR* and *KRAS* mutations are mutually exclusive, and Asian patients with NSCLC tend to have more *EGFR* mutations and thus fewer *KRAS* mutations [20]. The frequency of *KRAS G12C* mutations in Caucasians ranges from 35 to 45% [16, 18, 21, 22]. In our study, 29.5% of *KRAS* mutations in the mCohort were of the *G12C* subtype, which means that nearly 3% of these Chinese NSCLC patients harbored *KRAS G12C* mutations.

In Caucasians, *KRAS* mutations are more common in females and smokers [21–23]. Moreover, Dogan et al. and Osta et al. reported that *G12C* mutations were more common in women and those with a smoking history [17, 24]. By contrast, we found that male smokers more commonly harbored *KRAS* mutations, including *G12C* mutations, which is consistent with Guan et al. [25]. Although only a small proportion of *KRAS* mutation patients enrolled in the mCohort were diagnosed with stage I or II disease, *KRAS* mutation seems to be an early event that might drive lung cancer development [18, 21, 22, 25]. Furthermore, the *KRAS G12C* mutation might be a drug target in early stage lung cancer.

The prognostic role of *KRAS* mutations in NSCLC patients in early and advanced stages is becoming clear. Two studies enrolled surgically resected lung adenocarcinoma patients and found that those with *KRAS* mutation tumors had worse disease free survival and OS compared with wildtype patients [21, 22]. Even after excluding *EGFR* mutations, a significant survival difference persisted. A poor prognosis of *KRAS* mutation patients in advanced lung cancer stages has also been reported [17, 26]. Guan et al., from our institute, enrolled stage I to IV lung cancer patients. To eliminate bias of disease stage, patients were randomly paired, and *KRAS* mutations still predicted a poor prognosis [25]. Our results are consistent with the available data indicating a shorter OS for *KRAS* mutations, compared to wildtype tumors. However, the prognostic role of *KRAS G12C* mutations has been rarely reported. Nadal et al. showed that the *KRAS G12C* mutation was associated with poor outcomes in surgically resected lung adenocarcinoma and remained an independent prognostic marker for OS in multivariate analysis [22]. Svaton et al. indicated that patients with *G12C* mutations had shorter median OS compared to non-*G12C KRAS* mutation and wildtype patients (6.4 vs 10.3 vs 16.1 months, *P* = 0.01) [26]. In our study, more than 80% of the NSCLC patients exhibited advanced disease. The median OS of the *G12C* mutation and wildtype patients was 18.3 and 26.7 months, respectively (HR = 1.66, *P* = 0.007). The prognostic value of the *G12C* mutation was identified in Cox regression analysis. Although the *P-*value reached statistical margin, this may have been due to the small sample size of *KRAS* mutation patients in the iCohort. Patients with *KRAS* mutations could receive chemotherapy as standard treatment; some could choose immunotherapy with anti-PD-1/PD-L1 blockade. No other choices were available for clinicians to prescribe for these patients. Thus, detailed information regarding the treatment is not presented in Table 2. In general, the *KRAS G12C* mutation was a prognostic biomarker for poor OS in Chinese NSCLC patients.

Our study included the largest sample size of NSCLC patients harboring *KRAS* mutations thus far. However, it had a few limitations. First, we only had clinical and pathological data for the NSCLC patients from multiple centers and lacked survival data. Thus, the prognostic role of the *KRAS G12C* mutation in poor OS were taken from only one of the centers, namely, the iCohort. Second, in the iCohort, there were more stage IV patients in the *KRAS* mutation subgroup than in the wildtype subgroup, which may have affected the results where *KRAS* mutations were associated with a poor prognosis (*P* = 0.01). However, this result has been repeated in previously published studies. Similarly, although more patients had stage IV disease in the *G12C* subgroup than in the wildtype subgroup, the difference in their distribution did not reach statistical significance (*P* = 0.10). Thus, results regarding the prognostic roles of *KRAS* mutations and *G12C* mutation were reliable. Third, racial differences in the *KRAS G12C* mutation should be explored further in future studies.

## Conclusion

In general, our study identified that approximately 9% of Chinese NSCLC patients had *KRAS* mutations. Of these, nearly 30% harbored the *KRAS G12C* mutation subtype, which often occurred in male smokers. The *KRAS G12C* mutation predicted a poor OS, which could potentially be improved by specific *G12C* inhibitors in the future.

## Data Availability

The datasets used and analyzed during the current study are available from the corresponding author on reasonable request.

## Abbreviations

CI: Confidence interval
EGFR-TKIs: Epidermal growth factor-receptor tyrosine kinase inhibitors
HR: Hazard ratio
LCLC: Large cell lung cancer
*MEK*: MAP-ERK kinase
NGS: Next-generation sequencing
NSCLC: Non-small cell lung cancer
OS: Overall survival
